# Glass fiber reinforcement in PMMA dentures: Industrial vs. commercial fibers for enhanced physico-mechanical properties

**DOI:** 10.1016/j.jtumed.2026.03.006

**Published:** 2026-03-27

**Authors:** Mehmood Asghar, Shahab Ud Din, Muhammad Kaleem, Bushra Naureen, Nawshad Muhammad, Muhammad S. Zafar

**Affiliations:** a Department of Dental Materials, Army Medical College, National University of Medical Sciences (NUMS), Rawalpindi, Pakistan; b Department of Dental Materials, School of Dentistry (SOD), Shaheed Zulfiqar Ali Bhutto Medical University (SZABMU), G-8/3, Islamabad, Pakistan; c Department of Pharmaceutical Chemistry, Shifa College of Pharmaceutical Sciences, Shifa Tameer-e-Millat University, Islamabad, Pakistan; d Department of Dental Materials, Institute of Basic Medical Science, Khyber Medical University Peshawar, KPK, Pakistan; e Department of Clinical Sciences, College of Dentistry, Ajman University, Ajman, United Arab Emirates; f Center of Medical and Bio-allied Health Sciences Research, Ajman University, Ajman, United Arab Emirates; g Faculty of Dentistry, University of Jordan, Amman, Jordan

**Keywords:** Fiber–glass interface, Fracture toughness, Glass fiber reinforcement, PMMA, بولي ميثيل ميثاكريلات, تدعيم بالألياف الزجاجية, متانة الكسر, واجهة الألياف الزجاجية

## Abstract

**Objectives:**

This study was aimed at comparing the effects of incorporating pre-silane-treated commercial (StickNET) (Exp-I) and industrial-grade (Exp-II) glass fibers (GFs) into polymethyl methacrylate (PMMA) (control) on fracture toughness (FT), hydrolytic degradation, and micromorphology.

**Materials and methods:**

Specimens were prepared and tested according to the ISO 201795–1 (2013) guidelines for denture base materials. The FT of notched specimens (40 mm × 8 mm × 4 mm) was evaluated with a universal testing machine. Water sorption (WS), mass uptake, desorption, and solubility of the specimens (40 mm × 8 mm × 4 mm) were measured through drying, immersion in deionized water, and drying in a dry-heat oven at 38 °C. Micromorphological/chemical assessment of prepared and fractured specimens was performed with scanning electron microscopy (SEM) and energy dispersive X-ray spectroscopy (EDS).

**Results:**

Significant improvements in FT were observed after GF incorporation into PMMA. Exp-I showed the highest FT (3.96 ± 0.05 MPa m^1/2^) and was followed by Exp-II (3.94 ± 0.16 MPa m^1/2^) and the control (3.32 ± 0.18 MPa m^1/2^). Exp-I (22.09 ± 0.86 μg/mm^3^) and Exp-II (22.71 ± 2.49 μg/mm^3^) reinforced specimens had significantly lower WS (*p* < 0.05) than the control (25.65 ± 2.28 μg/mm^3^). The control exhibited the highest mean mass uptake (*p* < 0.05), whereas the desorption (*p* < 0.01) was highest for Exp-I at equilibrium. By contrast, Exp-I (1.22 ± 0.71 μg/mm^3^) and Exp-II (2.38 ± 0.45 μg/mm^3^) had higher solubility than the control (0.14 ± 0.06 μg/mm^3^). SEM surface imaging confirmed that both GFs had a woven design, and the fractured specimens in both groups revealed a brittle fracture type with void-free bonding between PMMA and GFs due to sufficient silane treatment.

**Conclusion:**

GF incorporation significantly increased the FT of PMMA, thus decreasing WS and increasing solubility. Morphologically, both unreinforced and GF-reinforced PMMA specimens exhibited a brittle fracture type, and GF incorporation resulted in void-free impregnation of the matrix. GF incorporation is recommended as a viable option for enhancing the FT of PMMA denture base resins.

## الملخص

**أهداف البحث:**

هدفت هذه الدراسة إلى مقارنة تأثير دمج الألياف الزجاجية المعالجة مسبقًا بالسيلان، سواء التجارية (StickNET) (المجموعة التجريبية الأولى) أو ذات الدرجة الصناعية (المجموعة التجريبية الثانية)، في راتنج بولي ميثيل ميثاكريلات (المجموعة الضابطة)، وذلك على متانة الكسر، والتحلل المائي، والخصائص الميكرو-مورفولوجية.

**طرق البحث:**

تم تحضير العينات واختبارها وفق إرشادات المنظمة الدولية للتقييس الخاصة بمواد قواعد أطقم الأسنان. وتم تقييم متانة الكسر لعينات ذات شق مسبق بأبعاد (40 مم × 8 مم × 4 مم) باستخدام جهاز الاختبار الشامل. كما تم قياس امتصاص الماء، وزيادة الكتلة، وفقد الكتلة بالتحرر (إزالة الامتصاص)، والذوبانية لعينات بالأبعاد نفسها من خلال التجفيف، ثم الغمر في ماء منزوع الأيونات، ثم إعادة التجفيف في فرن حراري جاف عند درجة حرارة 38°م. كما أُجري التقييم الميكرو-مورفولوجي والكيميائي للعينات المحضرة والمكسورة باستخدام المجهر الإلكتروني الماسح ومطيافية الأشعة السينية المشتتة للطاقة.

**النتائج:**

لوحظ تحسن ذو دلالة إحصائية في متانة الكسر بعد تدعيم راتنج بولي ميثيل ميثاكريلات بالألياف الزجاجية. فقد أظهرتلمجموعة التجريبية الأولى أعلى قيمة لمتانة الكسر (3.96 ± 0.05 ميغاباسكال·م^1/2^)، تلتها المجموعة التجريبية الثانية (3.94 ± 0.16 ميغاباسكال·م^1/2^)، ثم المجموعة الضابطة (3.32 ± 0.18 ميغاباسكال·م^1/2^). كما أظهرت العينات المدعمة بالألياف الزجاجية في المجموعة التجريبية الأولى (22.09 ± 0.86 ميكروغرام/مم³) والمجموعة التجريبية الثانية (22.71 ± 2.49 ميكروغرام/مم³) انخفاضًا ذا دلالة إحصائية في امتصاص الماء مقارنة بالمجموعة الضابطة (25.65 ± 2.28 ميكروغرام/مم³). وأظهرت المجموعة الضابطة أعلى متوسط لزيادة الكتلة، في حين كانت إزالة الامتصاص الأعلى في المجموعة التجريبية الأولى عند حالة الاتزان. وأظهرت المجموعتان التجريبيتان ذوبانية أعلى مقارنة بالمجموعة الضابطة؛ حيث بلغت في المجموعة التجريبية الأولى (1.22 ± 0.71 ميكروغرام/مم³) وفي المجموعة التجريبية الثانية (2.38 ± 0.45 ميكروغرام/مم³)، مقابل (0.14 ± 0.06 ميكروغرام/مم³) في المجموعة الضابطة. كما أكدت صور المجهر الإلكتروني الماسح أن كلا نوعي الألياف الزجاجية يمتلكان بنية منسوجة، وكشفت العينات المكسورة في كلتا المجموعتين عن نمط كسر هش مع ترابط خالٍ من الفراغات بين راتنج بولي ميثيل ميثاكريلات والألياف الزجاجية نتيجة المعالجة الكافية بالسيلان.

**الاستنتاجات:**

أدى دمج الألياف الزجاجية إلى زيادة معنوية في متانة الكسر لراتنج بولي ميثيل ميثاكريلات، مع انخفاض امتصاصالماء وزيادة الذوبانية. ومن الناحية المورفولوجية، أظهرت العينات غير المدعمة والمدعمة بالألياف الزجاجية من راتنج بولي ميثيل ميثاكريلات نمط كسر هش، كما أدى دمج الألياف الزجاجية إلى تشريب متجانس للمصفوفة خالٍ من الفراغات. ويوصى باستخدام تدعيم الألياف الزجاجية كخيار فعّال لتحسين متانة الكسر لراتنجات قواعد أطقم الأسنان المصنوعة من بولي ميثيل ميثاكريلات.

## Introduction

Polymethylmethacrylate (PMMA) resins are extensively used worldwide for fabricating denture bases, owing to their beneficial properties including low cost, ease of fabrication, satisfactory esthetics, and color-matching ability.^1^, ^2^, ^3^ Despite their clinically beneficial properties, PMMA-based resins have drawbacks such as low thermal conductivity, time-dependent hydrolytic degradation due to high water sorption (WS) and solubility, high coefficient of thermal expansion, inadequate hardness, flexural and impact strengths.^4^, ^5^, ^6^ Consequently, these materials are prone to fracture and require frequent repair or replacement.

The frequent fracture of PMMA dentures results from different types of forces, such as flexural and impact stresses.^1^ Repeated flexing and loading of acrylic dentures induce flexural fatigue, and lead to microcrack formation in regions of stress concentration. The fusion of these micro-cracks, coupled with their propagation, results in structural failure in the denture bases.^6^^,^^7^ By contrast, impact-related failures of PMMA-based dentures typically occur outside the oral cavity, such as from a sudden blow or accidental dropping.^6^^,^^8^

The mechanical properties of PMMA can be enhanced in various ways. One method is the replacement of PMMA with other denture base materials, such as epoxy resins and polyamides.^9^ Alternatively, PMMA can be co-polymerized with a rubber-based material (e.g., butadiene styrene) to enhance its flexibility and impact resistance.^8^ Incorporation of metallic wires or various types of fibers has also been suggested to strengthen PMMA-based dentures.^6^^,^^8^^,^^10^ However, their incorporation often negatively affects the esthetics and generates areas of stress concentration that compromise the denture's fracture resistance.^3^^,^^9^^,^^11^ The incorporation of carbon/graphite fibers into PMMA significantly increases the impact and flexural strengths of PMMA resins.^12^ However, their greyish appearance limits their clinical acceptance, because of esthetic concerns.^8^^,^^13^

White-colored ultra-high molecular weight polyethylene does not affect the esthetics of acrylic resins and has been shown to enhance their mechanical attributes.^3^^,^^9^ However, a substantial drawback of these fibers is the requirement for plasma pretreatment to ensure adequate adhesion between the fibers and the PMMA resin.^12^ Similarly, aramid fibers, despite their ability to significantly improve the fracture toughness (FT) and tensile strength of PMMA denture bases, are not commonly used because of their unappealing yellow color and the difficulty in evenly incorporating them into acrylic resin.^3^^,^^8^

The incorporation of glass fibers (GFs) is among the most effective methods to reinforce PMMA denture resins.^3^^,^^14^, ^15^, ^16^, ^17^, ^18^, ^19^, ^20^ GF considerably enhances the mechanical properties of PMMA without affecting optical properties or esthetics.^2^^,^^10^^,^^21^, ^22^, ^23^ Furthermore, GFs can be easily bonded to PMMA after silane treatment and therefore have the potential to reinforce PMMA denture base materials..^8^^,^^24^

GFs of various types (e.g., electrical (E)-type and silica (S)-type) and orientations (unidirectional or multidirectional/woven)^25^ have been used for PMMA reinforcement. E-type or S-type GFs have been incorporated in PMMA and yielded encouraging results.^8^^,^^12^^,^^14^ Woven GFs are more effective in improving the impact and flexural strengths of PMMA resins.^15^^,^^16^^,^^26^ However, several studies have reported superior mechanical properties of PMMA with incorporation of unidirectional GFs.^27^^,^^28^ A drawback with unidirectional GFs is that they only reinforce PMMA in a single plane. Furthermore, ensuring their uniform distribution within the PMMA matrix is challenging, and uneven “clumping” of these fibers significantly decreases strength.^8^^,^^12^^,^^14^ This problem can be avoided with the use of multidirectional, mesh-based GFs, because of their ease of incorporation and handling.^3^^,^^8^

We previously demonstrated the cost-effectiveness of industrial woven GFs compared with commercial-grade GFs (StickNET), and their ability to enhance flexural strength.^8^ We have also demonstrated that silane treatment of industrial GFs results in the same pattern of chemical bonding to PMMA as that in dental-grade GFs, according to Fourier transform infrared spectroscopy (FTIR) spectroscopy.^29^ However, information on the influence of this incorporation on FT and physical properties, such as WS, solubility, desorption, and mean mass uptake, is important to assess the clinical durability of PMMA denture bases.^3^

Hence, this study was aimed at evaluating and comparing the FT and physical properties of two types of industrial GF-reinforced heat-cured PMMA with commercial and unreinforced PMMA resins. The fracture toughness of the notched specimens was measured with a universal testing machine. Microscopic imaging of the intact and fractured specimens was performed using scanning electron microscopy (SEM). The relative elemental compositions of the fibers were evaluated with energy dispersive X-ray spectroscopy (EDS).

## Materials and Methods

### Materials

**Figure 1:**
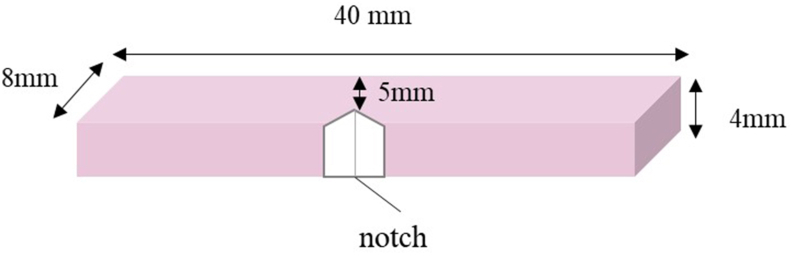
Specimen dimensions for fracture toughness measurement.

Heat-cured PMMA resin (powder and liquid) was obtained from MR Dental, UK. Commercial woven E-glass fibers (StickNET) were procured from StickTech, Finland, and industrial woven E-glass fibers were obtained from Iqbal Sons, Pakistan (Figure 1a,b). The A174 silane coupling agent (98 % (trimethoxysilyl)propyl methacrylate silane was obtained from Sigma Aldrich, USA. The control and experimental groups of this study are shown in Table 1.

**Table 1:** Study groups.

Control	Exp-I	Exp-II
Heat-cured PMMA	PMMA + StickNET	PMMA + Silane treatment + industrial-grade GFs

Sample preparation and testing were performed according to the ISO 201795-1 guidelines^30^ for denture base materials. Commercial StickNET GFs are pretreated with a silane-based coupling agent by the manufacturer. By contrast, industrial GFs require silane pretreatment before incorporation into PMMA.

### Silane treatment of industrial GFs

The technique for silane treatment of the industrial GFs has been described in detail in previous studies.^8^^,^^29^ The fibers were first cleaned by rinsing with distilled water to remove loosely bound contaminants. A subsequent brief acid etching process involved immersing the fibers in 1.6 M hydrochloric acid (HCl) solution for 60 s. This activated the glass surface by hydrolyzation of the siloxane bonds and increased the surface density of silanol groups. After a second rinse with distilled water to eliminate residual acid, the fibers were air-dried for 24 h to ensure complete removal of moisture, a critical aspect to ensure optimal silanation.

An 8 % (v/v) solution of silane coupling agent was then prepared in 98 % ethyl alcohol, and a catalytic amount of HCl was added to promote hydrolysis of the silane's alkoxy groups, thus facilitating their conversion to reactive silanols. The pretreated fibers were submerged in this solution for 24 h to allow formation of a uniform silane monolayer as the ethanol gradually evaporated.

The silanated fibers were then subjected to thermal curing at 100 °C for 24 h, to promote a condensation reaction between the silanol groups on the fiber surface and the silane. The fibers underwent a final distilled water wash to remove any unreacted silane or byproducts, and were incorporated into PMMA.

### Specimen preparation and measurement of FT

A gypsum mold measuring 40 mm × 8 mm × 4 mm was prepared by investing a highly polished stainless steel plate into a dental flask filled with freshly mixed type III dental gypsum. The plate was removed after the gypsum had set, and a thin coat of alginate-based separating medium was applied over the mold. Subsequently, a PMMA syrup was prepared with a powder-to-liquid ratio of 5/4 (w/v), and the StickNET and industrial-grade GFs were immersed for 30 s. The consistency of this “syrup” was selected to ensure optimal wetting of the fibers and therefore good adhesion between the GFs and the PMMA matrix. Afterward, the GFs were immediately incorporated into the PMMA resin in the doughy stage.

No fiber addition or silane treatment was performed in the control group. In the experimental groups (Exp-I and Exp-II), woven E-glass fibers (i.e., StickNET™ and industrial GFs, respectively) were sandwiched within the acrylic resin dough. The volume of the industrial GFs was adjusted to match that of the StickNET™ GFs (Exp-I). The thickness of the StickNET™ fibers was 0.06 mm, whereas that of the industrial fibers was 0.02 mm. Hence, two layers of StickNET™ GFs were incorporated in Exp-I, whereas six sheets of GFs were incorporated in Exp-II to equalize the volume. Heat-cured PMMA (Meadway Supercure, UK) mixing was performed with a powder-liquid ratio of 2.35 g/mL, then incorporated into the mold when the doughy stage was reached. Afterward, both halves of the flask were closed under hydraulic pressure.

After removal of the flask from the press, the PMMA resin was cured in a heat-curing apparatus at 70 °C for 2 h, then subjected to terminal boiling for 1 h at 100 °C. After the curing cycle was complete, the cured acrylic plate was retrieved from the flask, and four specimens (measuring 40 mm × 8 mm × 4 mm) were prepared from each plate with a slow-speed diamond saw (Isomet 4000 Linear) operating at 600 RPM. The specimens (n = 8) were then ground and polished with 600-grit metallographic paper under constant water cooling. Sample sizes for FT were selected based on previous studies, which used a sample size <6^31^^,^^32^ for reinforced and 3D printed PMMA denture base resins.

A notch 3 mm deep and 1 mm thick was made along the midline of prepared specimens, with a water-cooled saw (Figure 1). The notch was lubricated with a drop of glycerol and further sharpened with a sharp knife. Notched specimens were stored in a water bath at 37 °C for 7 days and then at 23 °C for 60 min before testing.

During testing, each specimen was dried and placed on UTM supports (AG20KNX Plus, Shimadzu, Japan) with the notch facing exactly opposite from the UTM load plunger, and the UTM supports were spaced 32 mm apart. The force was increased from 0 N at a displacement rate of 1 mm/min until the maximum load had passed, the crack had almost reached the opposite side of the specimen, or the current load was reduced to 5 % of the maximum load or fell below 1 N. The maximum stress intensity factor (K_max_) was measured with the following formula, as specified in ISO 20795–1 ^30^:

Kmax=10−3fPmaxlt(btht32)

where,

x = a/h_t_
P_max=_maximum load exerted on specimens (N)
a = crack length (mm)
a' = pre-crack length (mm)
h_t_ = height of specimen (mm)
b_t=_width of specimen

### Specimen preparation for measurement of water sorption, solubility, mass uptake, and desorption

Bar-shaped specimens (45 × 4 × 3 mm) were prepared as described in previous studies^33^^,^^34^ (n = 6). The sample sizes for WS and solubility were selected in strict accordance with ISO 20795–1:2013,^30^ which mandates a minimum of five specimens for flexural testing and WS protocols.

### Measurement of water sorption and solubility

The WS in μg/mm^3^ was measured with the following formula:

$${\;W}_{\text{sp}=\frac{m_{2-}m_{3}}{V}\;}$$

where m_2=_mass of specimen after immersion (μg)

m_3=_mass of reconditioned specimens (μg)
V = volume of specimen (mm^3^)

After the specimens had reached equilibrium during re-conditioning, water solubility (W_sl_) of immersed specimens (μg/mm^3^) was then recorded with the following formula:

$${\;W}_{\text{sl}=}\frac{m_{1-}m_{3}}{V}$$

where m_1=_conditioned mass of specimens (μg)

m_3=_re-conditioned mass of specimens (μg)
V = volume of specimens after immersion (mm^3^)

### Measurement of mean mass uptake

The cumulative water absorption of control, Exp-I, and Exp-II PMMA at various time points (i.e., 24 h to 21 days) was measured using a gravimetric method. Specimens (n = 6) measuring 45 × 4 × 3 mm^3^ were preconditioned in an oven (JSR, JSON 100, Japan) at 37 °C ± 1 °C for 15 days. Each specimen was removed from the oven and weighed on a calibrated microbalance (Sartorius, CP3245, USA) accurate to four decimal places. This initial weight was denoted W_0._ The specimens were then immersed in 100 mL distilled water and kept in glass screw top jars in a water bath (Memmert WNE 45, Germany) at 37 °C for 21 days.

Specimen weight was recorded at predetermined time intervals (every 24 h for 1 week, then at regular intervals until 21 days). The water bath temperature was reduced to 23 °C, 1 h before the specimens were weighed. Each specimen was removed from the solution, air-dried in air for 15 s, blotted with filter paper for 1–2 s to remove excess water from the surface, weighed, and then returned to the solution inside the water bath.

The mass uptake (%) at time t (s^½^) was calculated for each specimen with the following equation:

$$M_{t}=\frac{\left(W_{t}-W_{0}\right)}{W_{0}}\times 100$$

where W_0_ = initial weight (μg) at time 0.

W_t=_weight (μg) at time t
M_t=_mass uptake at time t

The data obtained from this experiment were plotted as mass uptake (%) versus the square root of time (s^1/2^).

### Measurement of mean desorption

After the 21-day immersion period, specimens were kept in a dry heat oven (JSR, JSON 100, Japan) at 37 °C ± 1 °C. The specimens were weighed at the same time points as those for mass uptake (i.e., every day for 1 week, followed by regular intervals up to 21 days) until equilibrium (W_d_) was reached. The desorption (%) was calculated and plotted against time (s^½^) to determine the desorption profile for each group.

### SEM imaging of the GFs and fractured specimens

SEM images of the GF meshes and PMMA specimens that had fractured during flexural strength testing were obtained, and their morphology and surface characteristics were assessed. The specimens were gold-sputtered under high vacuum to enhance their electrical conductivity, then observed under a scanning electron microscope (JEOL JSM-2300, Japan) at an accelerating voltage of 20 kV. Images for morphological evaluation were taken at magnifications of 50 × and 1000 × , whereas the images for fiber thickness were recorded at 10,000 × . The mean widths of the industrial and StickNET GFs were also recorded in ImageJ Software.

### EDS

Chemical analysis of the oxide compounds in the industrial (untreated and silane treated) and StickNET GFs (n = 3) was performed to evaluate their oxide composition. SEM was conducted with a JSM-6490A (JEOL, Japan) instrument equipped with an EDS analyzer at an accelerating voltage of 20 kV, with a probe current of 1 nA and a mean counting rate of 2600 counts per s (cps).

### Statistical analysis

Statistical analysis was performed with the Special Package for Social Sciences version 22 (SPSS 22). Comparisons of the FT values and oxide compositions among the control and experimental groups were performed with one-way analysis of variance (ANOVA) followed by post-hoc Tukey's test for intergroup comparisons. The significance threshold was set *p* < 0.05. The comparisons of mean fiber thickness were performed with one-way ANOVA.

## Results

### FT

**Figure 2:**
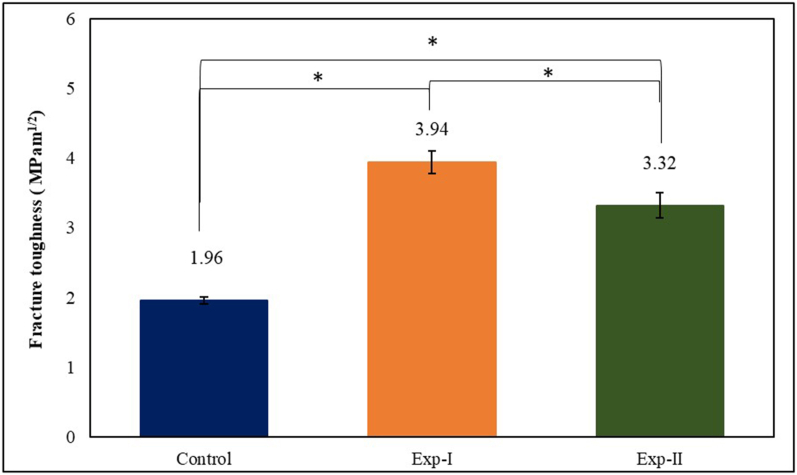
The Mean FT values of all the tested specimens.

Figure 2 shows the mean FT values for all tested specimens. Significant differences in the FT values of all three groups (*p* < 0.01) were determined with one-way ANOVA. Post hoc Tukey's test indicated a significant difference in mean FT values across all groups. StickNET-reinforced PMMA specimens (Exp-I) showed the highest FT and were followed by the industrial GF reinforced specimens (Exp-II), whereas the unreinforced (control) specimens had the lowest FT among tested specimens.

### Water sorption and solubility

The mean WS and solubility values for the unreinforced (control) and experimental (Exp-I and Exp-II) specimens are shown in Table 2. One-way ANOVA indicated significant differences in WS among the three specimen groups (P ≤ 0.01). Tukey's honest significant difference post hoc test indicated that the mean WS values were significantly lower (*p* < 0.05) in the experimental (Exp-I and Exp-II) PMMA groups than the unreinforced (control) group. However, no significant difference was observed in the mean WS values between Exp-I and Exp-II.

**Table 2:** Mean values of water sorption and solubility for control and experimental PMMA specimens.

Specimen	Water sorption (μg/mm^3^)	SD	Solubility (μg/mm^3^)	SD
Unreinforced (control)	25.65	2.28	0.14	0.06
StickNET reinforced PMMA (Exp-I)	22.09	0.86	1.22	0.71
Industrial GF reinforced PMMA (Exp-II)	22.71	2.49	2.38	0.45

One-way ANOVA indicated significant differences in W_sl_ among the specimen groups (*p* < 0.01). Moreover, post hoc Tukey's test indicated significant differences in mean W_sl_ among all three groups. The control specimens showed the lowest solubility, whereas Exp-II showed the highest solubility, followed by Exp-I specimens (Table 2).

### Mean mass uptake

**Figure 3:**
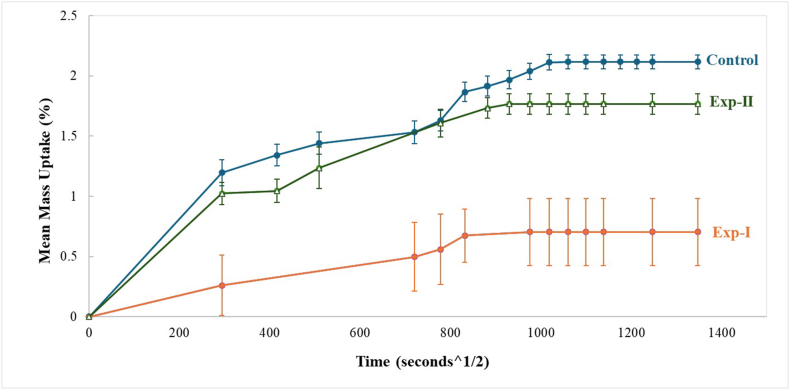
The time dependent change in the mean mass of control and experimental specimens during immersion.

The data for mean mass uptake were plotted as percentage mass uptake (y-axis) against the square root of time in seconds (x-axis), from which the mass uptake of the control and experimental PMMA groups was determined during a period of 21 days (Figure 3).

The control and experimental PMMA specimens reached equilibrium within 21 days (Figure 3) and showed significant differences among groups (ANOVA, *p* < 0.05). The control PMMA specimens had significantly higher mass uptake values than specimens from Exp-I and Exp-II at 24 h, 7, 11, and 21 days. However, no significant difference in mass uptake was observed between the control and Exp-I groups on the sixth day after immersion. A comparison between experimental PMMA groups indicated significantly higher values in Exp-II than Exp-I at 24 h, 7, 11, and 21 days.

### Mean desorption

**Figure 4:**
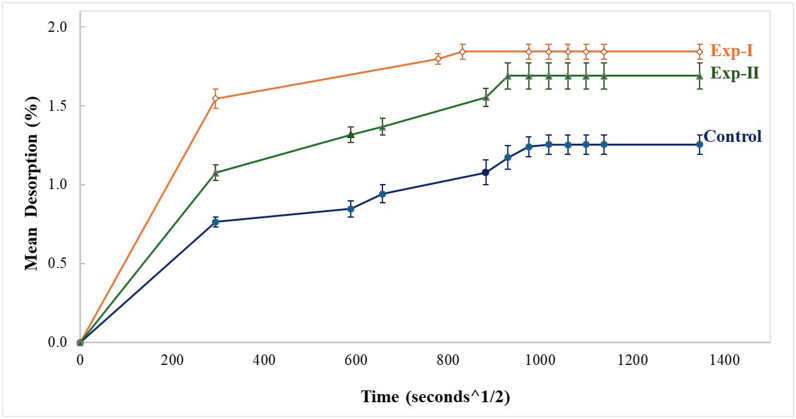
The mean desorption values for all the groups of PMMA specimens.

Figure 4 shows the mean desorption values for all PMMA specimen groups.

### Scanning electron microscope (SEM imaging

**Figure 5:**
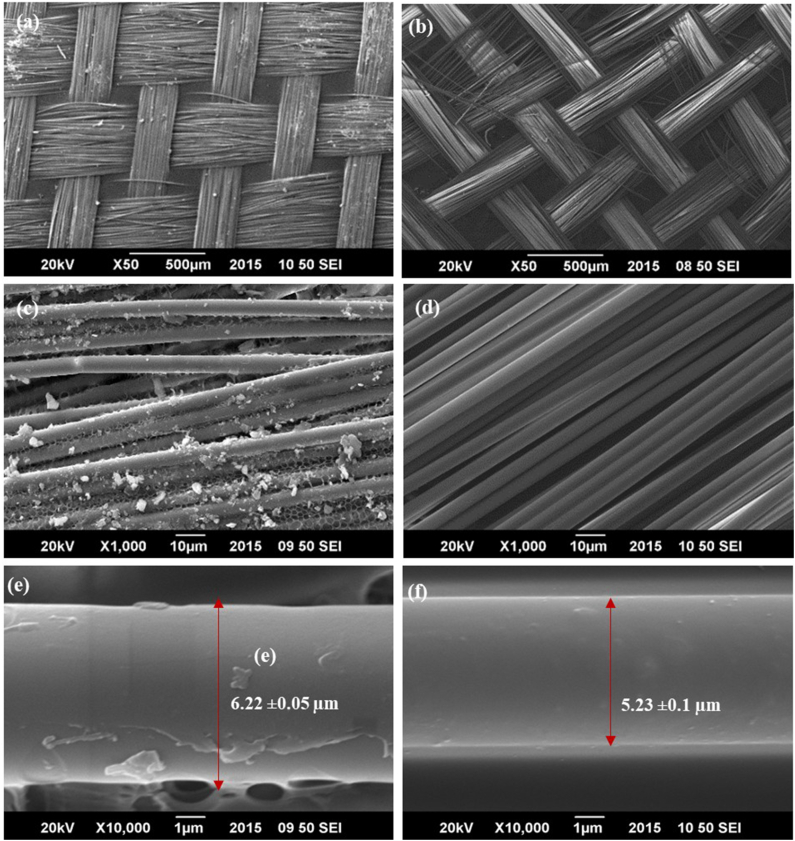
The Mean FT values of all the tested specimens.

The surface characteristics and weave pattern of StickNET and industrial GFs at 50 × (Figure 5a,b) and 1000 × (Figure 5c,d) were determined. Both StickNET and industrial GFs appeared woven, with the industrial GFs showing greater spacing between the weaves than StickNET. The mean thickness of the StickNET and industrial GFs, measured at 10,000 × , was 5.23 ± 0.11 μm and 6.22 ± 0.05 μm, respectively (Figure 5e,f).

**Figure 6:**
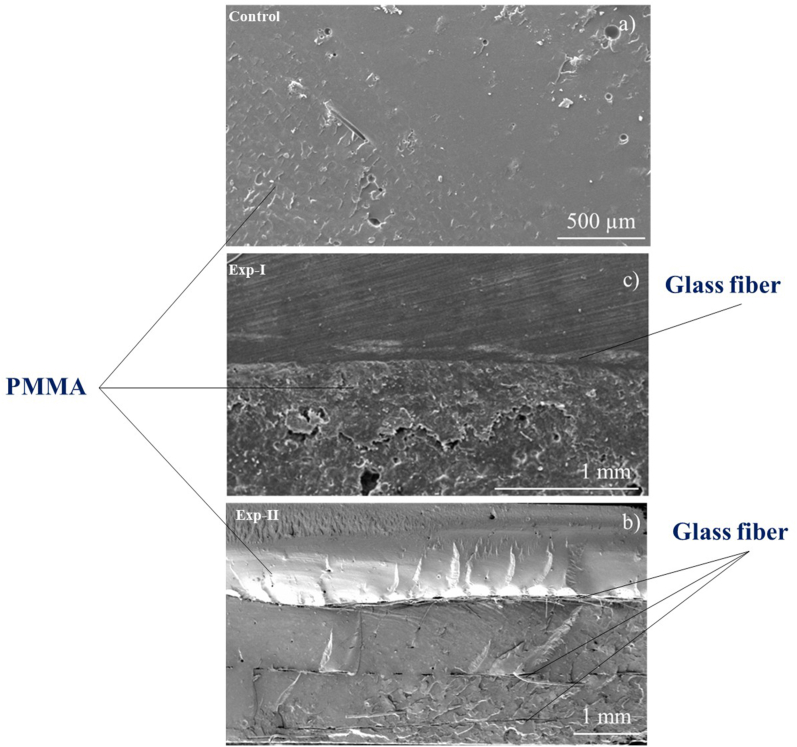
The representative cross-sectional SEM micrographs of the fractured control, Exp-I, and Exp-II specimens.

Figure 6 shows representative cross-sectional SEM micrographs of the fractured control, Exp-I, and Exp-II specimens.

### Energy dispersive X-ray spectroscopy (EDS)

**Figure 7:**
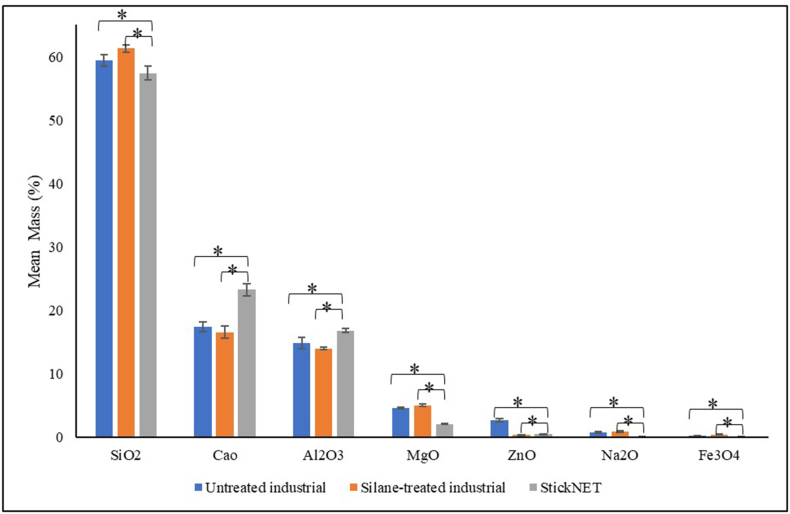
The results of the EDS analysis of StickNET, untreated and silane-treated industrial fibers.

Figure 7 shows the results of EDS analysis of the StickNET, untreated, and silane-treated industrial fibers. One-way ANOVA revealed significant differences in the mean oxide compositions of all compounds among all three groups. Furthermore, a pairwise comparison with post hoc Tukey's test indicated significant differences in composition between the pairs control–Exp-I and Exp-I–Exp-II, for all compounds in all three groups. Moreover, a single GF sheet was incorporated in Exp-I, whereas three sheets were incorporated in Exp-II. Consequently, the higher number of interfaces in Exp-II might have enhanced the solubility and affected long-term durability.

## Discussion

Herein, the FT values of PMMA resins were compared after incorporation of two E-type, woven GFs. The FT of a material is a measure of its ability to resist crack propagation in the presence of notches and inherent flaws. Removable dentures often incorporate a notch to accommodate the labial frenum, thus potentially introducing minute voids and structural flaws that can accelerate crack propagation. Hence, high FT is a highly desirable property in denture base materials.

We compared the effects of incorporation of two types of woven E-glass fibers procured from different sources (StickNET and industrial GFs) on the FT of PMMA with a three-point bending test on notched specimens. The StickNET-reinforced PMMA specimens (Exp-I) showed the highest FT, followed by the industrial GF-reinforced (Exp-II) and unreinforced (control) PMMA. These findings were consistent with those from previous studies indicating improvements in FT after fiber or filler reinforcement.^35^, ^36^, ^37^, ^38^

Yates et al.^38^ have investigated the effects of incorporation of various concentrations (1.5, 3, 5, and 7 wt.%) of E-glass fiber, zirconium oxide (ZrO_2_), and titanium oxide (TiO_2_) nanoparticles on the mechanical properties of PMMA. The 1.5 and 3 wt.% ZrO_2_, 1.5 wt.% TiO_2_, and all concentrations of E-glass fibers resulted in significantly greater FT compared with the control group (*p* < 0.05). Similarly, Franklin et al.^39^ have shown that the FT of PMMA is enhanced by a factor of 4–5 by reinforcement with various concentrations of glass flakes. However, in the current study, improvements in FT by factors of approximately 1.6 and 2 were observed for the industrial and StickNET GF reinforced specimens, respectively. The difference in FT values among studies may be because of differences in the type of reinforcement. Franklin et al.^39^ used 5–20 % w/w glass flakes, whereas in the current study, PMMA was reinforced with only 1.5 % v/v woven GFs. In another study, Geerts et al.^40^ have demonstrated a 38 % increase in the FT of PMMA after reinforcement with StickNET. However, we achieved much higher values of FT for Exp-I (3.93 ± 0.16) and Exp-II (3.32 ± 0.18 MPa m^1/2^), possibly because of differences in the dimensions of the tested specimens and the testing conditions.

Additional factors essential for improving the mechanical properties of GF-reinforced PMMA resins are the method, type, and concentration of the silane coupling agent used to chemically bind the GFs to the PMMA matrix.^41^ Uneven layering of GFs within PMMA might have been another reason for the inferior FT and flexural strength of Exp-I specimens. Three layers of industrial GFs were incorporated within the heat-cured PMMA. whereas, only a single layer of StickNET GFs was used in the preparation of reinforced specimens for the FT measurements of PMMA, which made even fiber incorporation and preparation of PMMA-GF composites relatively easier in Exp-I than Exp-II. To our knowledge, this study provides the first investigation of the effects of industrial GF reinforcement on the FT of PMMA.

The enhanced FT of the reinforced specimens was corroborated by examination of surface SEM images of the GFs and cross-sectional images of fractured PMMA specimens. The surface SEM images of both types of GFs confirmed their woven design. Furthermore, the absence of voids within the PMMA matrix indicated effective bonding between the resin and GFs and, more importantly, complete curing of the resin. These results were consistent with findings from Tomar et al.^42^ indicating a strong bond between GFs and PMMA matrix. Yates et al.^38^ have reported similar results indicating an absence of voids in the GF-reinforced PMMA matrix, thereby demonstrating effective impregnation of the GFs with PMMA.

In this study, the WS and solubility of unreinforced PMMA (control) were compared with two (StickNET and industrial) GF-reinforced PMMA specimens (Exp-I and Exp-II). The WS was significantly lower for GF reinforced specimens than unreinforced control specimens, in accordance with the findings of previous studies..^33^^,^^43^, ^44^, ^45^ Alhotan et al.^45^ have reported higher WS in unreinforced PMMA than GF-reinforced PMMA. However, WS showed a concentration-dependent increase in GF-reinforced PMMA, which became comparable to that in unreinforced PMMA at 7 wt.% concentration of GFs. Similarly, Cal et al.^46^ have reported significantly lower WS and dimensional changes in specimens reinforced with continuous and woven GFs than in unreinforced PMMA dibenzoylmethane (DBM).

Our results also showed that GF-reinforced PMMA specimens (Exp-I and Exp-II) had significantly higher solubility than the unreinforced (control) specimens, in agreement with findings from previous studies indicating that GF reinforcement of PMMA enhances its solubility.^33^^,^^47^ The greater solubility of the Exp-II than Exp-I specimens was attributed to the introduction of three layers of industrial woven GFs in Exp-II but only a single layer of GFs in Exp-I. Consequently, in Exp-II, three interfaces formed between the resin and the fibers, increasing the surface area for water to penetrate the composite and enhancing the solubility of the material. The formation of multiple interfaces in Exp-II specimens was also evident in the SEM images of transverse sections of the fractured specimens.

Although all groups remained within the ISO limits for WS, the solubility results provided a more complex picture. The industrial (Exp-II) group exceeded the maximum allowable limit of 1.6 μg/mm^3^. This finding was notable because high solubility implies leaching of unreacted monomers or other components from the material. Clinically, this possibility prompts concerns regarding biocompatibility, because leached components can irritate oral tissues and compromise long-term structural integrity, given that a material losing mass is likely to become brittle over time.

The one possible explanation pertains to the interface between the fiber and the resin matrix. StickNET fibers are manufactured with a pre-impregnated porous PMMA coating that effectively “melts” into the denture base and forms a seamless bond. The industrial fibers, which lacked this specialized dental-grade pretreatment, probably had poor wetting, thus creating microscopic voids at the fiber–matrix interface that served as channels for water ingress and monomer egress. Moreover, because a single GF sheet was incorporated in Exp-I, whereas three sheets were incorporated in Exp-II, the higher number of interfaces in Exp-II might have led to enhanced solubility and affected long-term durability.

However, the potential of industrial fibers should not necessarily be ruled out. Our findings simply suggest that these fibers cannot be used in their raw state. In future applications, treating these fibers with a silane coupling agent or manually pre-impregnating them with resin before mixing would probably seal the interface and decrease the solubility to safe, ISO-compliant levels.

SEM surface micrographs of the GFs also indicated a larger gap between the bi-directional weavings for the industrial GFs than the StickNET GFs. This aspect might have contributed to the higher FT in the Exp-I specimens than the control and Exp-II specimens, because these voids might have served as areas of stress concentration. The greater mean thickness of the individual StickNET GFs compared with the industrial GFs might have been another reason for the superior fracture resistance of the Exp-I specimens.

Furthermore, the cross-sectional SEM images of all fractured specimens indicated purely brittle fracture. These findings were consistent with the results of a previous study^48^ demonstrating a brittle type of fracture in pure PMMA and GF-reinforced PMMA specimens. Similarly, in a study on the fracture of GF-reinforced PMMA specimens by Gokul et al.^37^ the absence of fractures at the resin-GF interface in reinforced specimens indicated that both GFs achieved adequate bonding to the PMMA matrix, owing to sufficient silane treatment. Therefore, the use of silane-treated GFs, which are cost-effective and more readily available than commercial GFs such as StickNET, can effectively reinforce PMMA-based dentures and enhance their fracture resistance.

The difference in FT values between the GFs used in this study (StickNET and industrial) was also evaluated by comparison of their compositional differences through oxide compositional analysis (%) via EDS. Although the EDS results confirmed that both GFs were of the E-type, which is used most commonly for PMMA denture reinforcement, we observed statistically significant differences in the mean oxide compositions of untreated GF–StickNET GF and StickNET GF–industrial GF pairs. However, as expected, we observed no statistically significant difference in the mean oxide compositions of the untreated GF–industrial GF pair. The significant difference in the oxide composition between StickNET and industrial GFs might explain the difference in their FT values.

This study had several pertinent limitations. First, it was performed under laboratory conditions that did not completely mimic the oral environment. Moreover, cyclic loading (fatigue) simulation of the specimens was not performed to ensure closeness to the clinical scenario. Fiber reinforcement of PMMA resins was performed under laboratory conditions that might have differed from actual intra-oral conditions. Finally, the manual incorporation of fibers might have introduced variability with respect to industrial manufacturing. Hence, further clinical testing is needed to confirm the findings and correlate them with clinical settings. An important aspect for consideration in future studies is the biocompatibility of commercial and industrial GFs.

Moreover, future studies should focus on evaluating aging of the GF-reinforced PMMA. Specifically, tests such as dynamic fatigue loading and thermocycling should be performed to assess whether the fiber–matrix interface survives long-term physical and thermal stress. In addition, cytotoxicity assays will be mandatory to ensure that the leaching components do not pose a biological risk before any clinical trials are conducted. Finally, future studies should focus on improving the GF incorporation mechanism in PMMA, to achieve a solubility of industrial GF-reinforced PMMA specimens meeting ISO 20795-1 guidelines.

## Conclusion

Given the limitations of this study, we conclude that fiber reinforcement with woven E-type GFs can significantly enhance the FT of PMMA dentures. SEM micrographs of the fractured specimens showed void-free bonding between the resin and GFs and demonstrated a purely brittle fracture type. Finally, EDS analysis demonstrated that both the industrial and StickNET GFs had compositions typical of E-type GFs. Hence, we recommend industrial-grade E-type GFs as a cost-effective, readily available alternative to commercial, dental-grade woven GFs to improve the fracture resistance of PMMA dentures without affecting their esthetics and durability.

## Ethical approval

Not applicable.

## Authors' contributions

**MA:** Formal analysis, investigation, writing—original draft visualization. **SU:** Supervision, validation, writing—reviewing and editing, conceptualization. **MK:** Supervision, validation, writing—reviewing and editing, conceptualization, project administration, conceptualization. **BN:** writing—reviewing and editing, visualization, formal analysis, **NM:** writing—reviewing and editing, conceptualization, methodology. **MSZ:** validation, writing—reviewing and editing, conceptualization, project administration conceptualization. All authors have critically reviewed and approved the final draft and are responsible for the content and similarity index of the manuscript.

## Data availability

Data will be made available on request.

## Source of funding

This research did not receive any specific grant from funding agencies in the public, commercial, or not-for-profit sectors.

## Conflict of interest

The authors do not have any conflicts of interest to declare.

## References

[1] Aldegheishem A., AlDeeb M., Al-Ahdal K., Helmi M., Alsagob E.I. (2021). Influence of reinforcing agents on the mechanical properties of denture base resin: a systematic review. Polymers.

[2] Zafar M.S. (2020). Prosthodontic applications of polymethyl methacrylate (PMMA): an update. Polymers.

[3] Hassan M., Asghar M., Din S.U., Zafar M.S. (2019). Thermoset polymethacrylate-based materials for dental applications. Mat Biomed Eng Elsevier.

[4] Gad M.M., Fouda S.M., Al-Harbi F.A., Näpänkangas R., Raustia A. (2017). PMMA denture base material enhancement: a review of fiber, filler, and nanofiller addition. Int J Nanomed.

[5] Prajwala N., Kumar C.R., Sujesh M., Rao D.C., Pavani L. (2020). Denture base reinforcing materials-A review. IP Ann Prosthodont Restor dent..

[6] Alla R., Raghavendra K., Vyas R., Konakanchi A. (2015). Conventional and contemporary polymers for the fabrication of denture prosthesis: part I–overview, composition and properties. Int J Appl Decis Sci.

[7] Narva K.K., Vallittu P.K., Helenius H., Yli-Urpo A. (2001). Clinical survey of acrylic resin removable denture repairs with glass-fiber reinforcement. Int J Prosthodont (IJP).

[8] Asghar M., Ud Din S., Kaleem M. (2017). Effect of incorporating two different woven glass fiber reinforcent on the flexural strength of acrylic denture base materials. **Res Rev J Dent Sci** [Internet].

[9] Jagger D., Harrison A., Jandt K. (1999). The reinforcement of dentures. J Oral Rehabil.

[10] Vallittu P.K. (1996). A review of fiber-reinforced denture base resins. J Prosthodont.

[11] Abdulrazzaq Naji S., Jafarzadeh Kashi T.S., Behroozibakhsh M., Hajizamani H., Habibzadeh S. (2018). Recent advances and future perspectives for reinforcement of poly (methyl methacrylate) denture base materials: a literature review. J Dent Biomater.

[12] Uzun G., Hersek N., Tincer T. (1999). Effect of five woven fiber reinforcements on the impact and transverse strength of a denture base resin. J Prosthet Dent.

[13] Alla R.K., Raghavendra Swamy K., Vyas R., Konakanchi A. (2015). Conventional and contemporary polymers for the fabrication of denture prosthesis: part I–overview, composition and properties. Int J Appl Decis Sci.

[14] Gharoushi S.L.L., Vallitu P. (2009). Fibre-reinforced composites in clinical dentistry. Chin J Dent Res.

[15] Hari P.A., Mohammed H. (2011). Effect of glass fiber and silane treated glass fiber reinforcement on impact strength of maxillary complete denture. Ann Essences Dent.

[16] Kim S.-H., Watts D.C. (2004). The effect of reinforcement with woven E-glass fibers on the impact strength of complete dentures fabricated with high-impact acrylic resin. J Prosthet Dent.

[17] Alla Ss R., Alluri V., Ginjupalli K., Upadhya N. (2013). Influence of fiber reinforcement on the properties of denture base resins. J Biomater Biotech.

[18] Tacir I., Kama J., Zortuk M., Eskimez S. (2006). Flexural properties of glass fibre reinforced acrylic resin polymers. Aust Dent J.

[19] Schauperl Z., Ivanković L., Bauer L., Šolić S., Ivanković M. (2023). Effects of different surface treatments of woven glass fibers on mechanical properties of an acrylic denture base material. Int J Mol Sci.

[20] Mori H.V., Jadhav R., Sabane A., Patil A., Gachake A., Kalsekar B.G. (2023). An in vitro study comparing the impact and flexural strength of leucitone 199 denture base resin and conventional denture base resin enhanced with glass fibre mesh and polyethylene fibre mesh. Cureus.

[21] Mona K. (1999). Reinforcement of denture base resin with glass fillers. J Prosthodont.

[22] van Heumen C., Kreulen C.M., Bronkhorst E.M., Lesaffre E., Creugers N.H. (2008). Fiber-reinforced dental composites in beam testing. Dent Mater.

[23] Dudhe S.P., Thool P., Suganya P., Gupta S., Almudarris B.A., Bedi G. (2025). Comparing the flexural strength of PMMA enhanced with glass fibers and aluminum oxide: an in vitro study. J Pharm BioAllied Sci.

[24] Mohamed S.H., Ardelean L.C., Rusu L.-C. (2024). Advances in dentures - prosthetic solutions, materials and technologies.

[25] Krishna Alla R., Lakshmi Sanka G.S.S.J., Saridena D.S.N.G., Av R., Makv R., Raju Mantena S. (2023). Fiber-reinforced composites in dentistry: enhancing structural integrity and aesthetic appeal. Int J Dent Mat.

[26] Unalan F.D.I., Gurbuz O. (2010). Transverse strength of polymethylmethacrylate reinforced with different forms and concentrations of E-Glass fibres. Oral Health Dent Manag..

[27] Vojdani M., Khaledi A. (2006). Transverse strength of reinforced denture base resin with metal wire and E-glass fibers. J Tehnran Univ Medical Sci..

[28] Köroğlu A., Özdemir T., Usanmaz A. (2009). Comparative study of the mechanical properties of fiber-reinforced denture base resin. J Appl Polym Sci.

[29] Bhatti M.A., Ud Din S., Kaleem M., Hassan M., Naureen B. (2018). Comparison of the infra-red absorption peaks of untreated and silane treated glass fibers through fourier transform infra-red spectroscopy. Pak Oral Dent J.

[30] ISO. ISO 20795-1:2013(E) (2013).

[31] Hamza T., Wee A.G., Alapati S., Schricker S.R. (2004). The fracture toughness of denture base material reinforced with different concentrations of POSS. J Macromol Sci, Part A.

[32] Venkat R., Gopichander N., Vasantakumar M. (2013). Comprehensive analysis of repair/reinforcement materials for polymethyl methacrylate denture bases: mechanical and dimensional stability characteristics. J Indian Prosthodont Soc.

[33] Miettinen V.M., Vallittu P.K. (1997). Water sorption and solubility of glass fiber-reinforced denture polymethyl methacrylate resin. J Prosthet Dent.

[34] Miettinen V.M., Vallittu P.K. (1997). Release of residual methyl methacrylate into water from glass fibre-poly (methyl methacrylate) composite used in dentures. Biomater.

[35] Yilmaz C., Korkmaz T. (2007). The reinforcement effect of nano and microfillers on fracture toughness of two provisional resin materials. Mater Des.

[36] Vallittu P.K., Lassila V.P., Lappalainen R. (1994). Transverse strength and fatigue of denture acrylic-glass fiber composite. Dent Mater.

[37] Gokul S., Ahila S. (2018). Effect of E-glass fibers with conventional heat activated PMMA resin flexural strength and fracture toughness of heat activated PMMA resin. Ann Med Health Sci Res.

[38] Alhotan A., Yates J., Zidan S., Haider J., Silikas N. (2021). Assessing fracture toughness and impact strength of PMMA reinforced with nano-particles and fibre as advanced denture base materials. Mater.

[39] Franklin P., Wood D.J., Bubb N.L. (2005). Reinforcement of poly (Methyl methacrylate) denture base with glass flake. Dent Mater.

[40] Geerts G.A., Overturf J.-H., Oberholzer T.G. (2008). The effect of different reinforcements on the fracture toughness of materials for interim restorations. J Prosthet Dent.

[41] Aldabib J.M. (2021). Effect of silane coupling agent content on mechanical properties of hydroxyapatite/poly (methyl methacrylate) denture base composite. J Sci Perspect.

[42] Tomar P., Rana N., Sharma M., Bhargva M., Batra N.K. (2024).

[43] Miettinen V.M., Narva K.K., Vallittu P.K. (1999). Water sorption, solubility and effect of post-curing of glass fibre reinforced polymers. Biomater.

[44] Lassila L., Nohrström T., Vallittu P. (2002). The influence of short-term water storage on the flexural properties of unidirectional glass fiber-reinforced composites. Biomater.

[45] Alhotan A., Yates J., Zidan S., Haider J., Jurado C.A., Silikas N. (2021). Behaviour of PMMA resin composites incorporated with nanoparticles or fibre following prolonged water storage. Nanomaterials.

[46] Çal N.E., Hersek N., Sahin E. (1999). Water sorption and dimensional changes of denture base polymer reinforced with glass fibers in continuous unidirectional and woven form. Int J Prosthodont (IJP).

[47] Meriç G., Dahl J.E., Ruyter I. (2005). Physicochemical evaluation of silica-glass fiber reinforced polymers for prosthodontic applications. Eur J Oral Sci.

[48] Tomar P., Chandra Gope P. (2024). Effect of glass fiber and nylon fiber reinforcement on the mechanical and thermal properties of styrene butadiene rubber mixed PMMA denture base material. J Mech Behav Biomed Mater.

